# Occlusal features of 5-year-old Greek children: a cross-sectional national study

**DOI:** 10.1186/s12903-022-02303-1

**Published:** 2022-07-09

**Authors:** Sotiria Davidopoulou, Konstantinos Arapostathis, Elias D. Berdouses, Katerina Kavvadia, Constantine Oulis

**Affiliations:** 1 Department of Operative Dentistry, Dental School, Aristotle University of Thessaloniki, Thessaloniki, Greece; 2 Department of Paediatric Dentistry, Dental School, Aristotle University of Thessaloniki, Thessaloniki, Greece; 3 Department of Paediatric Dentistry, European University of Dubai, Dubai, United Arab Emirates; 4 Department of Pediatric Dentistry, European University, Nicosia, Cyprus; 5 Department of Paediatric Dentistry, National and Kapodistrian University of Athens, Athens, Greece

**Keywords:** Occlusion, Primary dentition, Non-nutritive oral habits

## Abstract

**Background:**

Occlusal characteristics of the primary dentition are crucial in predicting and determining permanent tooth alignment and occlusion. The aim of our study was to determine the occlusal characteristics of the primary dentition of 5-year-old children in Greece through a national pathfinder survey.

**Methods:**

A stratified cluster sample of 1222 5-year-old children was selected according to the WHO guidelines for national pathfinder surveys. Five occlusal traits were registered clinically in centric occlusion, separately for the left and right sides: sagittal relationships of the second primary molars and primary canines, overjet, overbite, crossbite, and maxillary and mandibular spacing.

**Results:**

Most children showed a flush terminal plane of primary second molars (44.8%), a class I primary canine relationship (52.2%) and normal overjet (46.4%), but a high prevalence of Class II canine relationship (25.6%) and overjet (37.8%) were also observed. A normal overbite was found in 40% of the children and 40% had a deep overbite. Spacing was apparent in both maxilla (71.1% of children) and mandible (56.4%). The prevalence of open bite and distal step molar relationship significantly rose in children with non-nutritive sucking habits.

**Conclusions:**

Νon-nutritive habits were associated to altered occlusal features. No sex significant differences were found in either the sagittal relationships of second primary molars and primary canines, or overjet, overbite, crossbite and spacing.

## Background

During the last decades, there has been a growing interest in the occlusion of primary dentition and an increased awareness of its role in the determination of permanent tooth alignment and occlusion. The key occlusal characteristics in primary dentition are space discrepancies, overbite, overjet, transverse (posterior crossbite) and sagittal (molar and canine relationship) deviations in the posterior region [1]. The presence of diastemas in the anterior region, a normal bite and overjet, a straight or mesial step molar relationship and a class I canine relatiοnship are predictors of favorable development of the permanent occlusion [2, 3]. Nevertheless, most epidemiological studies of occlusion features are devoted on the permanent dentition and the few studies on primary occlusion reveal that the most common orthodontic problems among preschool children are anterior crossbite, excessive overjet, Class ΙΙ malocclusion and posterior crossbite [4–7]. Such investigations, that study malocclusion and its variations in ethnic groups or local populations are of particular importance especially for the health delivery systems, to plan effective and sustainable preventive interventions and orthodontic services, targeted to the needs and requirements of the specific population.

The use of pacifiers and the digit or thumb sucking are common comforting behaviors, especially in young children. They are commonly referred as ‘non-nutritive sucking habits’ (NNSHs). In most children, the comforting habit stops as the child gets older, either under his own impetus or with parental or carers’ support [8]. The influence of non-nutritive sucking habits on the development of dental malocclusion is undoubtable [9, 10]. In general, they are associated with a higher prevalence of malocclusion in the primary dentition, that may include a Class II canine and molar relationship, anterior open bite and posterior crossbite, and increased overjet [11].

In Greece, national oral health pathfinder surveys have been conducted in 2004 and 2014, but only in the 2014 survey data regarding occlusal characteristics and non-nutritive sucking habits in preschool children were collected. These findings are presented in the present study regarding primary dentition occlusal features in 5-year-old children in relation to non-nutritive sucking habits and other parameters as sex and parental education.

## Methods

The aim of our study was to determine the occlusal characteristics of the primary dentition of 5-year-old children in Greece through a national pathfinder survey. A stratified cluster sample of 1222 5-year-old children was selected according to the World Health Organization guidelines for national pathfinder surveys [12]. The survey was performed in 24 sites, 15 urban and 9 rural, during the academic year 2013–2014. The sites were geographically distributed, in 6 mainland counties and 3 islands, in the Aegean and the Ionian Sea. Also, based on the same sites, the socio-economic backgrounds were achieved by sampling one urban and one rural community from the counties and islands and six sites from the two metropolitan areas. In each site two schools were randomly selected and at least 50 children (25 boys and 25 girls) were examined. Written informed consent was obtained from legal guardian(s) or from parents for each child to participate. All methods were carried out in accordance with relevant guidelines and regulations of the Declaration of Helsinki. An approval of the Ethics and Research Committee of the Dental School of the University of Athens (#201 and 202/21-03-2013) and a permission from the Ministry of Education of Greece for keeping sensitive personal data (#1131/10-10-2012) were obtained before the clinical examinations.

Prior to the survey, a meeting was organised in the Athens Dental School to train and calibrate 10 examiners. The procedure included a theoretical session with practical exercises (clinical images and extracted carious teeth), and a clinical session with oral examination. During the calibration process, the reference examiner plus each of the 10 trainees examined 20 children 3 times and the monitoring of calibration performance was possible in real time in order to achieve an acceptable level of > 85% in inter- and intra-examiner reliability, estimated by the weighted Cohen’s Kappa test [13]. Oral examinations were held in the classrooms of the selected schools, using artificial light using dental mirrors and the WHO periodontal probe.

Five occlusal traits were registered clinically in centric occlusion, separately for the left and right sides: (a) sagittal relationships of the second primary molars and primary canines, (b) overjet, (c) overbite, (d) crossbite, and (e) maxillary and mandibular spacing.

- The sagittal relationships of the second primary molars and primary canines were assessed in centric occlusion using Foster and Hamilton criteria [14].

- Primary second molar relationship: the relationship of the maxillary and mandibular second primary molars in the vertical plane. Flush terminal plane: the distal surfaces of upper and lower primary second molars are in one line with each other when the primary teeth are in occlusion. Distal step: the distal surface of lower primary second molar is distal to the distal surface of the primary upper second molar in occlusion. Mesial step: the distal surface of lower primary second molar is mesial to the distal surface of the upper primary second molar in occlusion. Unspecified: not possible to be recorded e.g. due to tooth loss.
- Primary canine relationship: Class I: the cusp tip of the upper primary canine is in the same vertical plane as the distal surface of the lower primary canine. Class II: the cusp tip of the upper primary canine tooth is mesial to the distal surface of the lower primary canine. Class III: the cusp tip of the upper primary canine is distal to the distal surface of the lower primary canine. Unspecified: not possible to be recorded e.g. due to tooth loss.

(b) Overjet (mm): measured from the mid-point of the labial surface of the most anterior lower central incisor to the mid-point of the labial surface of the most anterior upper central incisor, parallel to the occlusal plane. It was categorized as: reverse, normal (0–2 mm), increased (> 2 mm), zero when upper and lower incisal edges met edge to edge and unspecified.
(c) Overbite (mm): the vertical distance between the incisal edges of the upper and lower central incisors. It was categorized as: open bite, normal (0–2 mm), increased (> 2 mm), zero when upper and lower incisal edges met edge to edge and unspecified.
(d) Crossbite:

- Anterior crossbite. When one or more upper incisor teeth were palatal to the lower incisor teeth at maximum intercuspation.
- Posterior crossbite. When one or more lower posterior teeth (primary canines or primary molars) in any quadrant distal to the lateral incisor were placed buccal to the upper posterior teeth at maximum intercuspation. Posterior crossbite was recorded as unilateral, bilateral or none.

(e) Maxillary and mandibular spacing. Spaces between adjacent teeth in maxillary and mandibular arches were validated and each arch was estimated as with spaces and without spaces. Primary spaces, between maxillary lateral primary incisors and primary canines and mandibular primary canines and first primary molars, and secondary spaces were recorded.

Further data was collected through a structured questionnaire that was sent to children’s parents and was returned together with the signed consent form. The questionnaire provided information about the education level of both parents, and details about the presence or absence of oral habits and bottle-feeding practices. Regarding oral habits the following data were collected:

- Use of pacifier (yes-no) and frequency (during daytime, at sleep or both).
- Sucking of digit or thumb (yes-no) and frequency (during daytime, at sleep or both).

### Statistical analysis

Data were summarized as frequencies. Categorical variables were compared by Chi-square (χ^2^) test. All analyses were performed with SPSS v 20.0.0 statistical package. The significance level of all statistical tests was predetermined at p = 0.05.

## Results

In this cross-sectional study, occlusal features recorded at totally 1222 children (595 boys and 627 girls) of 5-years of age. Table 1 presents sample characteristics concerning sex, urban or rural area of residence and parental education. The distribution of different sagittal relationship of molars and canines, overjet, overbite, crossbite and spacing is presented in Table 2. The majority of children showed a flush terminal plane of primary second molars (44.8%), and a class I primary canine relationship (52.2%). Regarding overjet, most of the children (46.4%) had normal (0-2 mm) followed by children (37.8%) with increased (> 2 mm) overjet. Regarding overbite, 40.2% of the children demonstrated a normal overbite while the prevalence for increased overbite was almost similar (40.1%). Unilateral posterior cross bite was found in 8.8% of the children while anterior only in 4.8%. Spacing was apparent in both maxilla (71.1%of children) and mandible (56.4%). None of the occlusal features examined was related to sex. Moreover, no relation was observed between occlusal characteristics and area of residence, paternal, and maternal educational level.

**Table 1:** Demographic characteristics of the sample of 5-year-old children (sex, urban/rural residence area, parental education)

	N (%)
*Sex (n = 1222)*	
Male	595 (48.7%)
Female	627 (51.3%)
*Area of residence (n = 1222)*	
Urban	759 (62.1%)
Rural	463 (37.9%)
*Father educational level (n = 1222)*	
Up to lower secondary	193 (15.8%)
Upper secondary/non-university tertiary	738 (60.4%)
University	272 (22.3%)
Not known	19 (1.6%)
*Mother educational level (n = 1222)*	
Up to lower secondary	94 (7.7%)
Upper secondary/non-university tertiary	774 (63.3%)
University	342 (28%)
Not known	12 (1%)

**Table 2:** Prevalence of occlusal features in the 5 years old sample

Occlusal features		N (%)	Sex	
			Male	Female
Right primary second molar relationship n = 1222	Flush terminal plane	628 (51.4)	297 (47.3)	331 (52.7)
	Distal step	298 (24.4)	144 (48.3)	154 (51.7)
	Mesial step	285 (23.3)	149 (52.3)	136 (47.7)
	Unspecified	11 (0.9)	5 (45.5)	6 (54.5)
Left primary second molar relationship n = 1220	Flush terminal plane	664 (54,4)	320 (48.1)	344 (51.8)
	Distal step	268 (22.0)	122 (45.5)	146 (54.5)
	Mesial step	272 (22.3)	143 (52.6)	129 (47.4)
	Unspecified	16 (1.3)	8 (50.0)	8 (50)
Right primary canine relationship n = 1220	Class I	732 (60.0)	358 (48.9)	374 (51.1
	Class II	429 (35.2)	207 (48.3	222 (51.7
	Class III	49 (4.0)	26 (53.0)	23 (47.0)
	Unspecified	10 (0.8)	3 (30.0)	7 (70.0)
Left primary canine relationship n = 1219	Class I	771 (63.2)	368 (47.7)	403 (52.3)
	Class II	396 (32.5)	196 (49.5)	200 (50.5)
	Class III	43 (3.5)	26 (60.4)	17 (39.6)
	Unspecified	9 (0.7)	3 (33.3)	6 (66.7)
Overjet n = 1221	Reverse	20 (1.6)	10 (50.0)	10 (50.0)
	Normal (0–2 mm)	566 (46.4)	274 (48.4)	292 (51.6)
	Increased (> 2 mm)	461 (37.8)	237 (51.4)	292 (63.3)
	Zero	12 (1.0)	3 (25.0)	9 (75.0)
	Unspecified	162 (13.3)	70 (43.2)	92 (56.8)
Overbite n = 1220	Open bite	58 (4.75)	26 (44.8)	32 (55.2)
	Normal (0–2 mm)	490 (40.2)	233 (47.6)	257 (52.4)
	Increased (> 2 mm)	489 (40.1)	254 (51.9)	235 (48.1)
	Zero	23 (1.9)	10 (43.5)	13 (56.5)
	Unspecified	160 (13.1)	71 (44.4)	89 (55.6)
Anterior crossbite n = 1220	Yes	59 (4.8)	31 (52.5)	28 (47.5)
	No	1161 (95.2)	563 (48.5)	598 (51.5)
Posterior crossbite n = 1221	Unilaterally	107 (8.8)	51 (47.7)	56 (52.3)
	On both sides	15 (1.2)	8 (53.3)	7 (46.7)
	No	1099 (90.0)	536 (48.8)	563 (51.2)
Maxillary spacing n = 1219	No	352 (28.9)	168 (47.7)	184 (52.3)
	Yes	867 (71.1)	427 (49.3)	440 (50.7)
Mandibular spacing n = 1219	No	531 (43.6)	259 (48.8)	272 (51.2)
	Yes	688 (56.4)	336 (48.8)	352 (51.2)

The presence of sucking of digit or thumb was observed in 8.4% of children while the use of a pacifier in 3.5%. The results presented in Table 3 regarding non-nutritive sucking in relation to sex and parental education, indicate that although there was no significant relationship between sucking of digit or thumb and parental education, the use of pacifier was significantly correlated to both paternal (Chi-square = 8.147, p = 0.019) and maternal educational level (Chi-square = 6.584, p = 0.035). No correlation was observed to area of residence. According to our data, 23% of children performed the habit during the day, 49% during the night and 28% in both cases, with no statistical relation to sex or parental education. Moreover, 25% of children with the habit of pacifier, practiced it during the daytime, 50% during sleep at night and 25% both daytime and night with no statistical relation to sex or parental education.

**Table 3:** Distribution and prevalence of oral habits in relation to sex and parental education

		N (%)	Sex		Father educational level			Mother educational level		
			Male N (%)	Female N (%)	Up to lower secondary N (%)	Upper secondary/non-university tertiary N (%)	University N (%)	Up to lower secondary N (%)	Upper secondary/non-university tertiary N (%)	University N (%)
*Sucking of finger or thumb (n = 1200)*										
Yes	101 (8.4)		55 (54.4)	46 (45.6)	21 (20.8)	55 (54.4)	23 (22.8)	11 (10.9)	63 (62.4)	25 (24.8)
No	1099 (91.6)		526 (47.9)	573 (52.1)	164 (14.9)	674 (61.3)	247 (22.5)	77 (7.0)	702 (63.9)	313 (28.5)
*Use of pacifier (n = 1187)*										
Yes	42 (3.5)		18 (42.9)	24 (57.1)	11 (26.2)	27 (64.3)	3 (7.1)	7 (16.7)	27 (64.2)	8 (19.1)
No	1145 (96.5)		555 (48.5)	590 (51.5)	171 (14.9)	693 (60.5)	266 (23.2)	79 (6.9)	730 (63.8)	327 (28.6)
					Chi-square = 8.147, p = 0.019			Chi-square = 6.584, p = 0.035		

Table 4 presents the results regarding bottle feeding practices in relation to sex and parental education. Almost one third of the children continued the usage of a feeding bottle beyond the age of 3-years-old. No significant correlations to sex or parental education were observed, although children of high educated parents tended to quit earlier the non-nutritive habit.

**Table 4:** Distribution of bottle feeding practices by age in relation to sex, and parental education

N (%)		Sex		Father educational level			Mother educational level			
		Male N (%)	Female N (%)	Up to lower secondary N (%)	Upper secondary/non-university tertiary N (%)	University N (%)	Up to lower secondary N (%)		Upper secondary/non-university tertiary N (%)	University N (%)
Bottle feeding practices (n = 1181)										
Until 18 months	423 (35,8)	208 (49.2)	215 (50.8)	62 (14.7)	249 (58.9)	106 (25.1)	31 (7.3)	262 (61.9)		127 (30.0)
Until 3-year-old	392 (33.2)	186 (47.4)	206 (52.6)	61 (15.6)	239 (61.0)	90 (23.0)	23 (5.9)	250 (63.8)		116 (29.6)
Over 3-year-old	366 (31)	175 (47.8)	191 (52.1)	62 (16.9)	228 (62.3)	68 (18.6)	34 (9.3)	244 (66.7)		85 (23.2)

Table 5 presents data regarding occlusal features in relation to non-nutritive sucking habits. Both sucking of fingers and use of pacifier significantly altered bilateral second primary molar relationship, specifically by increasing bilateral distal step (Chi-square = 12.292, *p* = 0.056 and Chi-square = 12.734, *p* = 0.047, respectively). Sucking of fingers significantly increased class II bilateral canine relationship (Chi-square = 14.440, *p* = 0.006), overjet (Chi-square = 21.235, *p* < 0.001), and open bite (Chi-square = 19.788, *p* = 0.001). The use of pacifier significantly altered overbite by increasing open bite (Chi-square = 21.447, *p* < 0.001) and increased the maxillary and mandibular spacing (Chi-square = 15.587, *p* = 0.049). Our results did not reveal any correlations of occlusal features to bottle feeding practices.

**Table 5:** Distribution and Prevalence of occlusal features by oral habits in the 5 years old sample

Occlusal features			Sucking of finger or thumb		Use of pacifier	
		Total N (%)	Yes N (%)	No N (%)	Yes N (%)	No N (%)
Bilateral second primary molar relationship (N = 1200)	Flush terminal plane	538 (44.8)	38 (37.6)	500 (45.5)	16 (38.1)	514 (44.9)
	Distal step	211 (17.6)	24 (23.8)	187 (17.0)	11 (26.2)	199 (17.4)
	Mesial step	222 (18.5)	13 (12.9)	209 (19.0)	5 (11.9)	216 (18.9)
	Flush terminal plane-distal step	110 (9.2)	16 (15.8)	94 (8.6)	1 (2.4)	106 (9.3)
	Flush terminal plane-mesial step	75 (6.3)	8 (7.9)	67 (6.1)	7 (16.7)	69 (6.0)
	Distal-mesial step	25 (2.1)	1 (1.0)	24 (2.2)	1 (2.4)	23 (2.0)
	Unspecified	19 (1.6)	1 (1.0)	18 (1.6)	1 (2.4)	18 (1.6)
	Total	1200 (100)	101 (100)	1099 (100)	42 (100)	1145 (100)
			Chi-square = 12.292, p = 0.056		Chi-square = 12.734, p = 0.047	
Bilateral primary canine relationship N = 1198	Class I	625 (52.2)	41 (40.6)	584 (53.2)	18 (42.9)	601 (52.6)
	Class II	307 (25.6)	38 (37.6)	269 (24.5)	16 (38.1)	289 (25.3)
	Class III	29 (2.4)	1 (1.0)	28 (2.6)	0 (0.0)	28 (2.4)
	Asymmetry	235 (19.6)	20 (19.8)	215 (19.6)	8 (19.0)	223 (19.5)
	Unspecified	2 (0.1)	1 (1.0)	1 (0.1)	0 (0.0)	2 (0.2)
	Total	1198 (100)	101 (100)	1097 (100)	42 (100)	1143 (100)
			Chi-square = 14.440, p = 0.006			
Overjet N = 1199	Reverse	19 (1.5)	1 (1.0)	18(1.6)	0 (0.0)	19 (1.7)
	Normal (0-2 mm)	558 (46.5)	27 (26.7)	531 (48.4)	14 (33.3)	537 (46.9)
	Increased (> 2 mm)	454 (37.9)	57 (56.4)	397 (36.2)	20 (46.7)	430 (37.6)
	Zero	11 (1)	0 (0.0)	11 (1.0)	0 (0.0)	11 (1.0)
	Unspecified	157 (13.1)	16 (15.8)	141 (12.8)	8 (19.0)	147 (12.8)
	Total	1199 (100)	101 (100)	1098 (100)	42 (100)	1144 (100)
			Chi-square = 21.235, p < 0.001			
Overbite N = 1198	Open bite	55 (4.6)	13 (12.9)	42 (3.8)	8 (19)	46 (4.0)
	Normal	485 (40.5)	30(29.7)	455 (41.5)	13 (31)	469 (41.0)
	Increased	481 (40.6)	42 (41.6)	439 (40.0)	15 (35.7)	460 (40.2)
	Zero	21 (1.8)	2 (2.0)	19 (1.7)	1 (2.4)	19 (1.7)
	Unspecified	156 (13.0)	14 (13.9)	142 (12.0)	5 (11.9)	149 (13.0)
	Total	1198 (100)	101 (100)	1097 (100)	42 (100)	1143 (100)
			Chi-square = 19.788, p = 0.001		Chi-square = 21.447, p < 0.001	
Anterior crossbite N = 1198	Yes	58 (4.8)	8 (7.9)	50 (4.6)	1 (2.4)	56 (4.9)
	No	1140 (95.2)	93 (92.1)	1047 (95.4)	41 (97.6)	1087 (95.1)
	Total	1198 (100)	101 (100)	1097 (100)	42 (100)	1143 (100)
Posterior crossbite N = 1198	Unilaterally	106 (8.8)	10 (9.9)	96 (8.8)	5 (11.9)	98 (8.6)
	On both sides	14 (1.2)	2 (2.0)	12 (1.1)	1 (2.4)	13 (1.1)
	No	1077 (89.9)	89 (88.1)	988 (90.1)	36 (85.7)	1031 (90.2)
	Total	1198 (100)	101 (100)	1097 (100)	42 (100)	1143 (100)
Maxillary-mandibular spacing N = 1197	On both maxilla and mandible	673 (56.2)	56 (55.4)	617 (56.3)	26 (61.9)	635 (55.6)
	Maxilla yes-mandible no	177 (14.8)	16 (15.8)	161 (14.7)	9 (21.4)	169 (14.8)
	No	347 (29.0)	29 (28.7)	318 (29.0)	7 (16.7)	338 (29.6)
	Total	1197 (100)	101 (100)	1096 (100)	42 (100)	1142 (100)
					Chi-square = 15.587, p = 0.049	

## Discussion

This is the first study providing epidemiologic data on occlusal characteristics of preschool children in Greece. Thus, it is useful in understanding normal occlusion and various types of malocclusion of the primary dentition of Greek children. Moreover, results on occlusion combined with the results on children’s non-nutritive oral habits, may support the development of effective primary and secondary prevention programs to achieve the goal of occlusal harmony and function in the permanent dentition.

Our results presented no sex differences in either the sagittal relationships of second primary molars and primary canines, or overjet, overbite, crossbite and spacing in accordance with previous studies [15–17]. The present study showed that the majority of the children in the sample (44.8%) had a bilateral ‘flush terminal plane’ molar relationship, an observation consistent with that reported in several other populations [1, 18, 19]. However, much higher percentages have also been reported [5]. In our study population, bilateral mesial and distal step molar relationship present almost equal percentages in accordance with previous studies [4], while in other populations mesial step is reported in much higher percentages than distal step [16, 17, 20], and even predominate in several other studies [16, 21]. Such differences between populations may be attributed to ethnic predilection, as has been previously suggested [22, 23].

52% of children in our study, had a bilateral Class I canine relationship while almost 20% presented asymmetry, in accordance with the studies of Bervian at al [1] and Abu Alhaija and Qudeimat [16], in Brazilian and Jordanian schoolchildren respectively. Nevertheless, most previous studies reported higher prevalence of Class I canine relationship at least over 75% [4, 18, 19, 24].

Discussing other occlusal parameters, the prevalence of increased overjet was found to be 37.8% rather higher than what previous studies reported [4, 16, 18, 19]. Moreover, increased overbite has also been detected in high prevalence (40%) in our study population in accordance with the study of Talebi et al. [24], while other studies reported lower prevalence of increased overbite [4, 18]. Both observations may be attributed to different methodology since in our study increased overjet was defined as over 2 mm while in the other studies over 3 mm.The anterior crossbite prevalence in the present study was found to be 4.8% much higher than previously reported in other study populations [4, 18, 19]. Regarding posterior crossbite, 8.8% of children presented a unilateral posterior crossbite while 1.2% of them on both sides. Results on posterior crossbite in other populations vary from zero to 13% [4, 7, 16, 18, 23].

Spacing in the present study was reported in 71% and 56.4% of the children, for the maxilla and mandible respectively. In more detail, 56.2% of children presented spacing in both arches. Our results coincide with the results of Hegde et al. [19], while other studies reported higher prevalence of spacing [7, 16].

Malocclusion is a developmental condition. In most of the cases it is caused by moderate distortions of normal development and it results through a complex interaction among multiple factors that influence growth and development [25]. Transition to discrepancies from primary to permanent dentition has led to an increased awareness of the role of primary dentition characteristics in the establishment of permanent occlusion [18]. Regarding Greek population, recent published data [26] regarding 12- and 15-years-old children, acquired in the same National Pathfinder survey of 2014, revealed a high prevalence of increased overjet (41.5% and 30.3% for 12- and 15-years old respectively) and overbite (48% and 33.2% for 12- and 15-years old respectively) as well, in accordance with our results for 5-years old children, a finding maybe indicating a prevalent occlusion pattern for Greek children. Another finding is that about 50% of 12-years-old were recorded with a class I Angle classification in Mylopoulou study [26] which is in accordance with our results where over 60% of children demonstrated a flush terminal plane or a bilateral mesial step for second primary molars and also over 50% of them, a class I primary canine relationship which consist favorable characteristics to develop a class I relation in permanent occlusion [2, 3].

Sucking behaviors are considered normal in infants and young children and derive mainly from the physiologic need for nutrients. They include nutritive sucking behaviors, consisting of breast- and bottle-feeding, and non-nutritive sucking, such as pacifier or finger sucking. Although such behaviors are considered normal in infants, the longer duration of the habit is associated to certain consequences on occlusion characteristics [27]. In the primary dentition, pacifier and digit sucking both present varying risks of developing features of malocclusions. In our study the prevalence of finger sucking and use of pacifier was 8.4% and 3.5% respectively, independent of sex, in a relevant agreement with equivalent studies in other populations [27, 28]. Moreover, we observed a correlation between father and mother educational level and prolonged use of pacifier. There is limited published research regarding who and what influences a mother’s decision to give a pacifier to her child but the association between lower mother education level and pacifier use is supported by previous studies [29, 30]. The above arise the need for better comprehension of the etiology of finger sucking and prolonged use of pacifiers through well designed studies. Moreover, educational programs and support for parents are necessary to diminish the side effects of such behaviors. Several studies have reported the association between non-nutritive oral habits and malocclusion, although most of them associated habits to malocclusions on mixed or permanent dentitions. Our results indicated that finger sucking and the use of pacifier significantly increased bilateral distal step molar relationship and open bite, while finger sucking also significantly increased Class II canine relationship and overjet, in accordance with previous studies [8–10, 31].

## Conclusions

The present study provides new insight into the occlusal pattern and spacing of primary dentition of Greek schoolchildren. No sex significant differences were found in either the sagittal relationships of second primary molars and primary canines, or overjet, overbite, crossbite and spacing. Although the majority of children demonstrated a flush terminal plane of primary second molars, a class I primary canine relationship and normal overjet, a tendency for higher Class II canine relationship, and increased prevalence of overjet and overbite were also observed in our study population. Spacing was more apparent in upper arch although in lower prevalence than in other study populations. Νon-nutritive habits were associated to altered occlusal features.

## Data Availability

Due to privacy, the datasets generated during and/or analysed during the current study are available from the corresponding author on reasonable request.

## Abbreviations

NNSHs: Non-nutritive sucking habits
WHO: World Health Organization

## Abbreviations

NNSHs: Non-nutritive sucking habits
WHO: World Health Organization
